# Tumor necrosis factor-α mediated inflammation versus apoptosis in age-related hearing loss

**DOI:** 10.3389/fnagi.2022.956503

**Published:** 2022-09-07

**Authors:** Ting Wu, Jiamin Zhou, Jingjing Qiu, Yuwan Song, Wentao Guo, Limei Cui, Xicheng Song, Yan Sun

**Affiliations:** ^1^Department of Otolaryngology Head and Neck Surgery, Yantai Yuhuangding Hospital, Qingdao University, Yantai, China; ^2^Shandong Provincial Clinical Research Center for Otorhinolaryngologic Diseases, Yantai, China

**Keywords:** presbycusis, TNF-α, NF-κB, inflammation response, age

## Abstract

An almost universal phenomenon occurring during aging is a state of chronic, low-grade, sterile inflammation. Inflammation is a crucial contributor to various age-related pathologies and natural processes in aging tissues. Tumor necrosis factor-α (TNF-α), a master regulator of the immune system, plays an important role in the propagation of inflammation. Recent research has found correlations between hearing loss and markers such as TNF-α. However, the intrinsic molecular mechanism by which TNF-α influences aging individuals’ increased risk of hearing loss remains unclear. In this study, we found that TNF-α expression gradually increased with age in DBA/2J mice. We then used recombinant TNF-α to upregulate TNF-α levels in House Ear Institute-Organ of Corti 1 (HEI-OC1) cells and found that low concentrations of TNF-α could activate the nuclear factor kappa B (NF-κB) transcriptional response to mediate hair cell survival, while high concentrations of TNF-α could activate the Caspase-3 cascade to mediate hair cell apoptosis, which preliminarily confirmed that a TNF-α mediated signaling pathway plays an important role in the pathogenesis of age-related hearing loss.

## Introduction

Presbycusis, also termed age-related hearing loss (ARHL), is progressive, irreversible, and symmetrical bilateral neuro-sensory hearing loss associated with aging caused by the limited repair capacity of sensorineural tissues in the cochlea (Keithley, 2020; Wang and Puel, 2020). The occurrence of ARHL seriously affects the elderly’s language communication, which decreases their quality of life and is a major contributor to social isolation and cognitive decline in the elderly (Panza et al., 2015; Mudar and Husain, 2016; Jayakody et al., 2018). Approximately one in five people over the age of 50 has imperfect hearing, and almost half of those aged over 65 years have hearing difficulties (Bowl and Dawson, 2019).

Aging is a progressive decline or loss of tissue and organ function over time caused by the gradual accumulation of deleterious biological changes. Pro-aging mechanisms include senescence-associated inflammation (Franceschi et al., 2018): many individuals become especially susceptible to chronic-inflammation-induced sensorineural hearing loss as they age. Recently, it has been suggested that there is an inflammatory presence in the cochlea (Watson et al., 2017). However, little is known about the exact mechanism of presbycusis and its relationship with inflammation. Tumor necrosis factor-α (TNF-α), which is mainly produced by monocytes and macrophages, can promote cell survival by activating the nuclear factor kappa B (NF-κB) pathway and promotes cell apoptosis by mediating the Caspase-3 pathway. TNF-α is believed to be involved in a variety of inflammatory diseases, including neurodegenerative diseases, such as Alzheimer’s disease (AD) (Decourt et al., 2017) and Parkinson’s disease (PD) (Wang et al., 2016), cancer (Yi et al., 2018), and diabetes (Akash et al., 2018). A characteristic of aging is the presence of chronic low-grade inflammation, which is typified by elevated concentrations of TNF-α (Franceschi et al., 2017). The polymorphisms of genes encoding inflammatory mediators such as TNF-α have been reported to contribute to the incremental risk of hearing impairment in elderly Japanese people (Uchida et al., 2014). In the aging cochleae, high levels of TNF-α have been observed (Lyu et al., 2020), and TNF-α is implicated in regulating the initiation and progression of noise-induced hearing loss (Fuentes-Santamaría et al., 2017). Moreover, *in vivo* loss-of-function analysis demonstrated that TNF-α was a factor that worsened cochlear inflammation (Satoh et al., 2003) and clinical research suggested that there was an increased TNF-α level in sudden sensorineural hearing loss (Yoon et al., 2019). However, the molecular mechanism underlying TNF-α’s increased risk of ARHL remains unclear.

Laboratory animals are useful when investigating ARHL because of their short lifespans and well-defined genetics. DBA/2J mice are an ARHL animal model, in which the hearing loss begins at approximately 3 weeks of age, with deterioration to total deafness by 3 months of age (Yang et al., 2015). Thus, the present study aimed to explore the expression changes of TNF-α, and inflammation and apoptosis-related factors, in the process of hearing loss in an ARHL mouse model, and to investigate the mechanism of TNF-α mediated inflammation and apoptosis signaling pathway in the pathogenesis of ARHL. Furthermore, an House Ear Institute-Organ of Corti 1 (HEI-OC1) inner ear cell model was used to explore the TNF-α concentration-mediated regulation of the survival and death of inner ear hair cells.

## Materials and methods

### Mouse preparation

DBA/2J mice were originally purchased from the Model Animal Research Center (Shanghai SLAC Laboratory Animal Co., Ltd., China) and then relocated to the Affiliated Yantai Yuhuangding Hospital of Qingdao University for breeding in specific pathogen free animal rooms. The room temperature was maintained at 24 ± 1^°^C and sufficient food and water were provided. The animal studies were conducted in accordance with the principles set forth in the Guide for the Care and Use of laboratory animals of the Affiliated Yantai Yuhuangding Hospital of Qingdao University, and were approved by the Institutional Animal Use and Care Committee of the Affiliated Yantai Yuhuangding Hospital of Qingdao University. A total of 50 DBA/2J mice aged 2–8 weeks old were used in this study.

### Auditory-evoked brainstem response

Auditory-evoked brainstem response was measured at various intervals (2, 4, 6, and 8 weeks) in the DBA/2J mice. Eight mice were selected for each week of age. A computer-aided evoked potential system (Intelligent Hearing Systems, Miami, FL, United States) was used to test mice for ABR thresholds. The amplified brainstem responses were averaged and displayed on a computer screen. A plug-in earphone was inserted into the external auditory canal of DBA/2J mice to stimulate short pure tones at different frequencies (8, 16, and 32 kHz). The frequency intensity started from 80 dB, and then gradually decreased. The minimum stimulus intensity of wave III was used as the auditory threshold (wave III disappeared last, so the ABR threshold was determined by wave III).

### Distortion-product otoacoustic emission

To test the function of the outer hair cells (OHC) of mice at 2, 4, 6 and 8 weeks, we used the IHS Smart EP 3.30 and USBez Software (Intelligent Hearing Systems) for distortion-product otoacoustic emission (DPOAE) measurement, which was conducted with pure tones from 6 to 35 kHz. Frequencies were acquired with an f2: f1 ratio of 1:22. The stimuli were presented from the lowest to the highest frequencies tested.

### Hematoxylin and eosin staining

Six mice were selected at the age of 2, 4, 6, and 8 weeks. The cochlea was excised, immersed in a fixative containing 4% paraformaldehyde in phosphate-buffered saline (PBS) solution for 1 day, and decalcified in 10% EDTA for 3–5 days. Sections (5 mm) mounted on glass slides were counterstained in hematoxylin and eosin (H&E). Hair cells, spiral ganglia neurons (SGNs), the stria vascularis (SV), and inflammatory cells were observed under a light microscope (OLYMPUS DP73, Tokyo, Japan). The mean value in four sections from a basal turn of each animal’s cochlea was defined as the width of the SV. The width of the SV and the average SGNs density was quantified using ImageJ software (NIH, Bethesda, MD, United States).

### Quantitative real-time reverse transcription PCR (qRT-PCR) verification

Samples of total RNA were reverse transcribed into cDNA using a Total-Transcriptome cDNA Synthesis Kit (AG11707, Accurate Biology, China) according to the manufacturer’s instructions. Quantitative real-time polymerase chain reaction measurements were performed using the StepOnePlus fluorescence quantitative PCR instrument (Applied Biosystems, Foster City, CA, United States) and a SYBR Green qPCR Mix according to the manufacturer’s instructions. The data were analyzed using the 2^–ΔΔ^*^CT^* method (Livak and Schmittgen, 2001), and the results were represented as relative expression levels, *Actb* (encoding actin) was the reference gene. The information on the primers used is shown in Table 1.

**TABLE 1:** The information on the primers used of qRT-PCR.

ID	Sequence
TNF-α-F	5′-GGATTACAAGGGATTACACAGGC-3′
TNF-α-R	5′-GCTACGACGTGGGCTACAG-3′
IL-6-F	5′-CTGCAAGAGACTTCCATCCAG-3′
IL-6-R	5′-AGTGGTATAGACAGGTCTGTTGG
NF-κB-F	5′-GCTGCCAAAGAAGGACACGACA-3′
NF-κB-R	5′-GGCAGGCTATTGCTCATCACAG-3′
c-FLIP-F	5′-GGATTACAAGGGATTACACAGGC-3′
c-FLIP-R	5′-CTGGTACTCCATACACTGGCT-3′
Caspase-3-F	5′-ATGGAGAACAACAAAACCTCAGT-3′
Caspase-3-R	5′-TTGCTCCCATGTATGGTCTTTAC-3′
Caspase-8-F	5′-TGCTTGGACTACATCCCACAC-3′
Caspase-8-R	5′-TGCAGTCTAGGAAGTTGACCA-3′
Actin-F	5′-GGCTGTATTCCCCTCCATCG-3′
Actin-R	5′-CCAGTTGGTAACAATGCCATGT-3′

### Western blotting

Cochlear tissue was ground with Radioimmunoprecipitation assay (RIPA) buffer (Beyotime, Jiangsu, China) containing phosphatase inhibitors (Roche, Basel, Switzerland) and phenylmethylsulfonyl fluoride (PMSF) (Beyotime). Total protein samples were separated by electrophoresis in 15% SDS polyacrylamide gels (Servicebio, China) and electrotransferred to a nitrocellulose membrane (Millipore, Billerica, MA, United States). The membrane was subsequently blocked with 5% non-fat milk for 1 h. Afterward, the membrane was incubated at 4°C overnight with the following primary antibodies: anti-NF-κB (1:2000 dilution) (P65, Santa Cruz Biotechnology Inc., Santa Cruz, CA, United States), anti-Caspase-3 (1:1000 dilution) (D3R6Y, Cell Signaling Technology Inc., Danvers, MA, United States).

Next day, the membrane was washed three times with Tris-buffered saline-Tween 20 (TBST) buffer, incubated with corresponding horseradish peroxidase-conjugated secondary antibody for 1 h at room temperature and then washed with TBST buffer three times. The immunoreactive proteins on the blots were visualized using the ECL reagent (Sparkjade Biotechnology, China) and detected using a chemiluminescence imaging system (ChemiScope 6200 Touch; Clinx Science Instruments Co., Ltd., Shanghai, China). The protein bands were quantified by detecting the gray values using ImageJ software.

### Immunohistostaining

A time course of immunostaining of Caspase-3 and NF-κB was carried out on DBA/2J mice. The time point selected was 4 weeks. After dewaxing, the cochlear tissues were subjected to heat induced antigen retrieval in citrate buffer (0.01 mol/L, pH 6.0), washed three times in 1 × PBS at room temperature for 5 min, and permeabilized with 3% H_2_O_2_ for 10 min. The slides were then blocked with 3% bovine serum albumin (BSA) and 3% goat serum at 37^°^C for 1 h and stained with rabbit anti-cleaved Caspase-3 antibody (1:250 dilution) (D3R6Y, Cell Signaling Technology Inc., Danvers, MA, United States) or NF-κB antibody (1:50 dilution) (P65, Santa Cruz Biotechnology Inc., Santa Cruz, CA, United States) at 4^°^C overnight. Following a thorough wash with 1 × PBS for 5 min, the sections were immersed in Goat anti-rabbit IgG(H + L) HRP (1: 400 dilution) (Sparkjade, China)and Goat anti-mouse IgG(H + L) HRP (1: 400 dilution) (Absin, China) at 37^°^C for 30 min, and then counterstained with 4 Detection Kit (ZLI-9018 DAB kit, ZSGB-BIO, China) for 15 min and with Harris hematoxylin (Sigma, China) for 1 min at room temperature. The slides were sealed using neutral gum and examined under a scanning confocal microscope.

### Cell culture

House Ear Institute-Organ of Corti 1 cells were cultured under permissive conditions (33°C, 10% CO_2_) in high-glucose Dulbecco’s Eagle’s medium (DMEM; gibco, China) containing 10% fetal bovine serum (FBS; gibco, China) without antibiotics as described previously (Kalinec et al., 2003).

### Cell viability quantification

Cell Counting Kit-8 (CCK-8) (DOJINDO, Kumamoto, Japan) reagent was used to examine cell viability according to the manufacturer’s instructions. HEI-OC1 cells were collected and counted, and the concentration was adjusted to 1.0 × 10^5^ cells/mL. The cells were then seeded on 96-well flat bottom plates (100 μL per well), incubated overnight for attachment to the substrate, and then treated with TNF-α at different concentrations (0, 1, 2.5, 5, 10, 20, and 40 ng/mL). After TNF-α treatment (12, 24, 36, or 48 h at 33°C, 10% CO_2_), 10 μL of CCK-8 reagent was added to each well and incubated for 1 h. Absorbance at 450 nm was detected using a plate reader.

### Cell transfection

House Ear Institute-Organ of Corti 1 cells were inoculated into 6-well plates, and the cell density was controlled at 30–50%. The cells were cultured in an incubator for 24 h. Liquid A comprised 1.25 pmol of different small interfering RNAs (siRNAs) (TNF-α siRNA-475, TNF-α siRNA-340, TNF-α siRNA-197, negative control, and fluorescent control) dissolved in 25 μL Opti-MEM solution (Gibco, United States). Liquid B comprised 0.75 μL of Lipofectamine 3000 (Thermo Fisher scientific, United States) was dissolved in 25 μL of Opti-MEM solution. Liquid A was mixed with liquid B gently and incubated at room temperature for 20 min. The 50 μL mixture was added to each well of HEI-OC1 cells, and continued in culture for 72 h. The information for the siRNAs used is shown in Table 2.

**TABLE 2:** The information on the primers for the siRNAs used.

ID	Sequence
TNF-α siRNA-475	5′-GCAUGGAUCUCAAAGACAATT-3′
	5′-UUGUCUUUGAGAUCCAUGCTT-3′
TNF-α siRNA-340	5′-CCCAAAGGGAUGAGAAGUUTT-3′
	5′-AACUUCUCAUCCCUUUGGGTT-3′
TNF-α siRNA-197	5′-GGAACUGGCAGAAGAGGCATT-3′
	5′-UGCCUCUUCUGCCAGUUCCTT-3′

### Statistical analysis

*ANOVA* was used to analyze the comparisons of ABR threshold and DPOAE amplitudes, the levels of gene transcription, the levels of protein expression, the widths of SV, the density of SGNs and the number of inflammatory cells were analyzed by *t*^2^-test. *P* < *0.05* was considered to be significant. Error Bars represent the standard deviation (SD) from the mean.

## Results

### Characterization of hearing and cochlea morphological changes in DBA/2J mice

**FIGURE 1:**
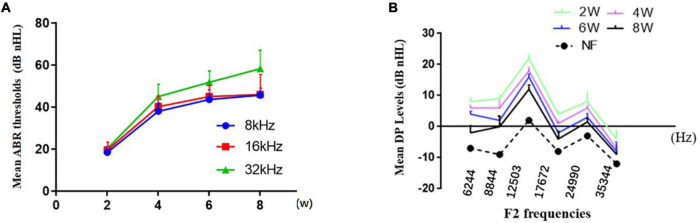
Characterization of hearing loss in DBA/2J mice. **(A)** The mean ABR thresholds increased from 2 to 8 weeks of age at different pure tone stimulus frequencies. **(B)** Decrease in DPOAE amplitudes with time.

Overall, the mean ABR (Figure 1A) thresholds increased with from 2 to 8 weeks of age at of 8, 16, or 32 kHz, and the level at 32 kHz was the highest at each time point. The mean ABR thresholds of the DBA/2J mice at stimulus frequencies of 8, 16, and 32 kHz were 43.6, 32.8, and 51.8 dB at 6 weeks of age, which basically agreed with the results of a previous study (Yang et al., 2015). We observed that DPOAE amplitudes declined with from 2 to 8 weeks of age (Figure 1B), indicating functional impairment of OHC.

**FIGURE 2:**
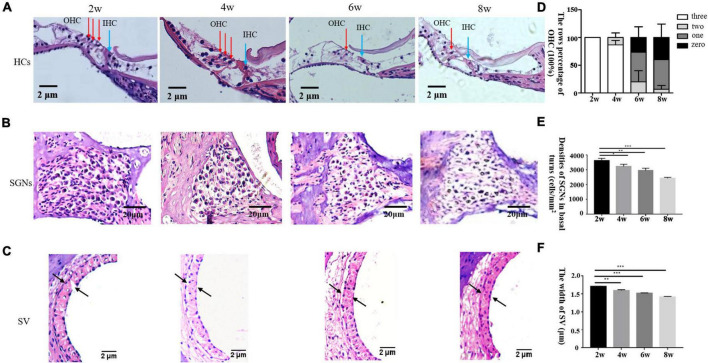
**(A–C)** Morphological changes in the cochlea after H&E staining in DBA/2J mice. OHC mean outer hair cell (red arrow), IHC means inner hair cell (blue arrow) × 200. Panel **D** shows the rows percentage of OHC (100%). Panels **E,F** show the statistical analysis of DBA/2J SGNs density and the SV width of mice at different weeks of age (*P < 0.05, **P < 0.01, ***P < 0.001).

Pathological examination by H&E staining (Figure 2) revealed progressive hair cell loss and the degeneration of SGNs in the cochleae of the DBA/2J mice. Both OHCs and inner hair cells (IHCs) were intact at 2 weeks of age. OHC loss was evident at 6 weeks of age, whereas IHCs persisted. Moreover, the density of SGNs was normal at 2 weeks of age, and decreased gradually from 4 to 8 weeks of age. The widths of the SVs are gradually decreased, becoming markedly narrowed.

### Characteristics of inner ear inflammation and apoptosis in DBA/2J mice

**FIGURE 3:**
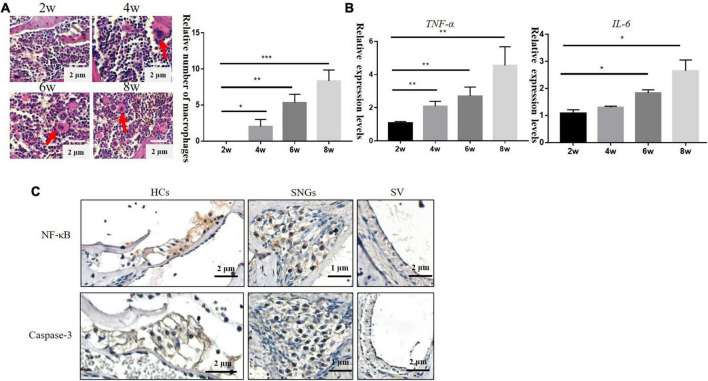
**(A)** Inflammatory cells in the lateral wall of the cochlea of DBA/2J mice increased with age, which were mainly macrophages (×200). **(B)** Relative mRNA expression levels of *Tnf-a* and *IL-6.* **(C)** Immunohistochemical: the location expression of NF-κB and Caspase-3 in the cochlea of DBA/2J mice at 4 weeks (*P < 0.05, **P < 0.01 ***P < 0.001).

With the loss of hair cells and SGNs in the inner ear, numbers of cochlear inflammatory cells in the cochlea wall also increased gradually, mainly by macrophage infiltration (Figure 3A). TNF-α is involved in systemic inflammation, the relative mRNA expression level of *Tfn-a* (encoding TNF-α) and *IL-6* increased progressively over time in DBA/2J mice (Figure 3B). TNF-α can mediate inflammation and apoptosis signaling pathways. Subsequently, immunohistochemistry was used to detect the localization of inflammatory and apoptotic factors. The results demonstrated that NF-κB and Caspase-3 were mainly located on hair cells and SGNs, with few located on SVs (Figure 3C).

**FIGURE 4:**
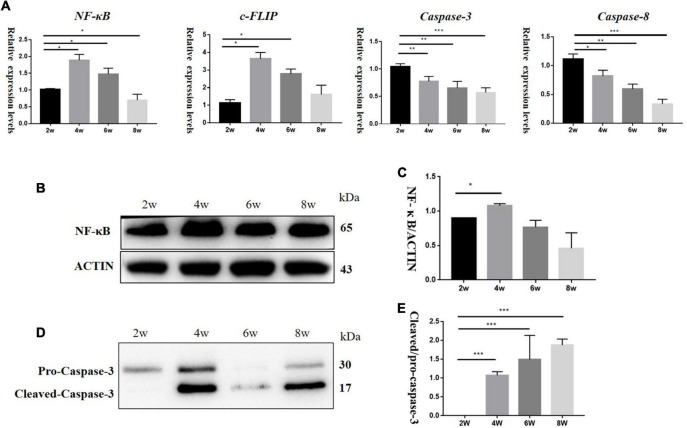
**(A)** It showed mRNA relative expression levels of *Nf-kb*, *C-flip*, *Casp3*, and *Casp8* in DBA/2J mice at 2,4, 6, and 8 weeks of age. **(B,D)** Protein levels of NF-κB and Caspase-3 in DBA/2J mice aged 2, 4, 6, and 8 weeks. **(C,E)** Semi-quantitative analysis of NF-κB and Caspase-3 protein levels (*P < 0.05, **P < 0.01, ***P < 0.001).

To explore the effect of TNF-α changes on inflammation and apoptosis in DBA/2J mice, we detected the mRNA levels of *Nf-kb*, *C-flip* (encoding CASP8 and FADD like apoptosis regulator, also known as C-Flip), *Casp3* (encoding Caspase-3), and *Casp8* (encoding Caspase-8) at the age of 2, 4, 6, and 8 weeks. The results showed that the relative mRNA expression levels of *Nf-kb* and *C-flip* also increased, with the highest expression at 4 weeks and then began to decline. The relative mRNA expression levels of *Casp3* and *Casp8* were higher at 2 weeks of age and then decreased (Figure 4A). Western blotting (Figures 4B,D) showed that the NF-κB levels were the highest at 4 weeks, and then decreased (Figure 4C), while the levels of pro-Caspase-3 protein gradually increased (Figure 4E).

### The survival of House Ear Institute-Organ of Corti 1 cells decreased as tumor necrosis factor-α concentration treatment

**FIGURE 5:**
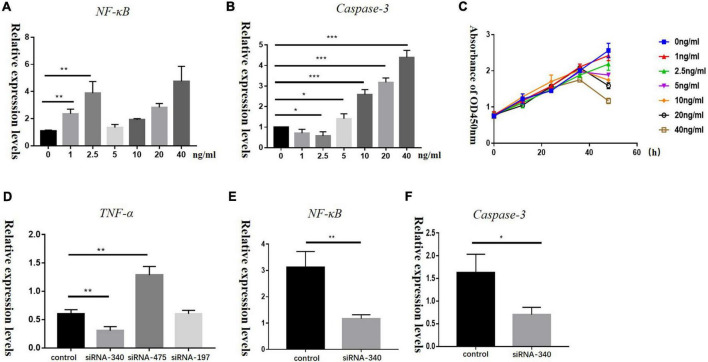
Panels **A,B** show the relative mRNA expression levels of *Nf-kb* and *Casp3* in HEI-OC1 cells treated with different concentrations of recombinant TNF-α (0, 1, 2.5, 5, 10, 20, and 40 ng/mL) for 72 h, (*P < 0.05, **P < 0.01, ***P < 0.001). **(C)** A CCK-8 assay was used to detect the proliferation of HEI-OC1 cells treated with recombinant TNF-α for 12, 24, 36, and 48 h, OD: optical density. Panels **D–F** represent relative mRNA expression levels of *Tnf-a*, *Nf-kb*, and *Casp3* in HEI-OC1 cells treated with siRNA-TNF-α for 72 h in control and blank groups, respectively (*P < 0.05, **P < 0.01).

To determine if TNF-α concentrations could influence the proliferation of HEI-OC1 cells, we treated HEI-OC1 cells with TNF-α at different concentrations (0, 1, 2.5, 5, 10, 20, and 40 ng/mL) for different times (12, 24, 36, and 48 h). Low concentration of TNF-α could increase NF-κB levels, while Caspase-3 and Caspase-8 levels barely changed. High concentrations of TNF-α increased both NF-κB and Caspase-3 levels in HEI-OC1 cells (Figures 5A,B). CCK-8 assays (Figure 5C) showed that a high concentration of TNF-α induced decreased proliferation and increased apoptosis of HEI-OC1 cells. The relative mRNA expression levels of *Nf-kb* and *Casp3* in the group transfected with TNF-α siRNA were significantly lower than those in the control group (Figures 5D–F).

## Discussion

In this study, we verified the characteristics of early onset hearing loss in DBA/2J mice. Hearing loss started in the high frequency region and was mainly manifested morphologically by hair cell loss, decreased SGN density, and a narrowed SV, which was basically consistent with previous studies. Thus, DBA/2J mice could be used as the animal model of senile deafness in this study.

Aging and age-related diseases share some basic mechanistic aspects that largely converge on inflammation. During aging, chronic, sterile, low-grade inflammation contributes to the pathogenesis of age-related diseases. Several investigations have shown that chronic inflammation plays an important role in the initiation and progression of damage to the inner ear (Masuda et al., 2012; Menardo et al., 2012; Verschuur et al., 2014) and presbycusis arises in the inner ear (Fischer et al., 2020; Keithley, 2020; Tawfik et al., 2020). Macrophages are always present in the lateral wall of the cochlea and are activated in response to various insults (Tan et al., 2016). A mild, chronic, systemic inflammatory response is thought to activate cochlear macrophages (Fischer et al., 2020). Several lines of evidence have revealed two cell types of the macrophage lineage in the lateral wall of the cochlea: “cochlear macrophages” in the spiral ligament and “perivascular macrophage-like melanocytes (PVM/Ms)” in the stria vascularis (Zhang et al., 2012). Numbers of cochlear macrophages were increased in lipopolysaccharide-induced cochlear inflammation (Bae et al., 2021). Apparently, increased numbers of macrophages can be observed in the lateral wall of the cochlea in 8-week-old DBA/2J mice compared with those in 2-week-old mice. This might indicate that chronic inflammation exists in the cochlea of DBA/2J mice, leading to the activation and recruitment of macrophages.

The inner ear was previously assumed to be an “immune-privileged” organ because of the existence of its tight junction-based blood-labyrinth barrier (BLB) in the stria vascularis (Shi, 2016). The barrier shields the inner ear from blood-born toxic substances and selectively passes ions, fluids, and nutrients to the cochlea, playing an essential role in the maintenance of cochlear homeostasis. Breakdown of the normal interactions between components of the BLB is observed in a wide range of pathological conditions, including conditions caused by inflammation and ageing. The normal function of the stria vascularis is also critical to maintain the ionic gradients and endcochlear potential (EP) required for sensory hair cell transduction (Zhang et al., 2012). Dysfunction of the stria, including the intrastromal fluid-blood barrier, is considered an etiology of a number of hearing disorders, including ARHL (Zhang et al., 2015). Inflammatory factor-induced hearing disorders are hypothesized to be associated with disrupted vascular integrity in the SV (Trune and Nguyen-Huynh, 2012). In our study, we found significant narrowing of the SV in 8-week-old DBA/2J mice, which might indicate damage. We speculated that chronic inflammation in the mouse cochlea might damage the integrity of the SV, leading its narrowing.

The role of chronic inflammation in hearing loss in ARHL and the related mechanisms are still unclear. TNF-α, which plays an important role in the propagation of inflammation *via* the activation and recruitment of immune cells through its receptor TNF receptor 1 (TNFR1), is associated with a variety of inflammatory diseases (Decourt et al., 2017; Akash et al., 2018; Cruceriu et al., 2020). In general, a cell’s life-or-death fate is mainly governed by two modules, survival or apoptosis. We discuss the signaling pathway of TNF-α, which can direct its signals down the survival and apoptosis modules (Varfolomeev and Vucic, 2018). Engagement of TNFR1 by TNF-α results in the sequential assembly of a membrane-bound primary signaling complex (complex I) that drives survival gene activation and the assembly of a secondary cytoplasmic complex (complex II) that mediates cell death (Micheau and Tschopp, 2003). NF-κB, the core protein of the survival module, regulates cell survival by inducing the expression of numerous anti-apoptotic genes. Caspase-3 is an apoptotic executioner caspase of the apoptosis module. When TNFR1 signaling to NF-κB is compromised, it can be diverted to initiate cell death. In general, members of the caspase family proteases jointly inhibit NF-κB activation, providing efficient pro-apoptotic behavior.

In our ARHL animal model, DBA/2J mice aged 8 weeks exhibited increased expression of the gene encoding the proinflammatory cytokine TNF-α compared with that at 2 weeks. TNF-α is mainly produced by monocytes and macrophages; therefore, these results were consistent with the presence of inflammatory infiltration. With the increase in TNF-α levels, the expression levels of the mRNAs encoding NF-κB and c-FLIP also increased, with the highest expression being observed at 4 weeks, after which it declined. The mRNA levels of *Casp3* and *Casp8* were higher at 2 weeks of age than at 4 weeks of age, and then decreased. In general, activation of TNFR1 does not induce death but instead triggers a robust pro-survival response. This response is suppressed unless some cell death checkpoints, which occur upon induction of pro-survival genes by NF-κB transcription factors, are disrupted. If NF-κB is blocked, the c-FLIP expression level diminishes and Caspase-3 undergoes auto-catalysis to initiate apoptosis. Based on the aforementioned results, we hypothesized that the downstream pathway activated by TNF-α signaling changed from the pro-inflammatory cell survival pathway to the cell destruction pathway before and after the death of inner ear cells.

Experimental studies have revealed that most cells survive at a low dose of TNF-α, while high doses of TNF-α induce apoptosis (Wajant et al., 2003). By simulating the dynamic behavior of the TNF-α modulated signaling network, Li et al. (2016) revealed that the critical dose of TNF-α can modulate the balance between cell survival and apoptosis. HEI-OC1 cells are derived from the auditory organ of a transgenic mouse. These cells have been used to investigate apoptotic pathways, senescence, and inflammatory responses (Kalinec et al., 2003). In the present study, the concentration of activated Caspase-3 remained low and the HEI-OC1 cell survived under a low dose of TNF-α. However, when the TNF-α dose increased beyond the critical dose (about 5 μg/mL), the HEI-OC1 cells driven into an apoptotic state, with a high concentration of activated Caspase-3. Interestingly, the concentration of activated NF-κB remained high under a high dose of TNF-α in our study.

A cell exposed to an inflammatory stimulus has several potential fates: survival, production of inflammatory cytokines and possibly proliferation; non-inflammatory or even anti- inflammatory apoptotic cell death; or pro- inflammatory programmed cell death (Galluzzi et al., 2018). In general, cell death can be both a consequence and a cause of inflammation, which can be difficult to distinguish. Under a high dose of TNF-α, HEI-OC1 cells undergo apoptosis, accompanied by membrane permeabilization and the release of pro-inflammatory cell contents. This could provide a reasonable interpretation of the experimental results.

In summary, TNF-α levels increased gradually with age in DBA/2J mice. Low concentrations of TNF-α can activate the NF-κB transcriptional response to mediate hair cell survival, while high concentration of TNF-α can activate the Caspase-3 cascade to mediate hair cell apoptosis. The TNF-α-mediated inflammatory signaling pathway plays an important role in the pathogenesis of ARHL.

## Data availability statement

The original contributions presented in this study are included in the article/supplementary material, further inquiries can be directed to the corresponding authors.

## Ethics statement

This animal study was reviewed and approved by the Institutional Animal Use and Care Committee of the Affiliated Yantai Yuhuangding Hospital of Qingdao University.

## Author contributions

TW: writing—original draft preparation. JZ and JQ: methodology and data curation. YuS and WG: find the relevant knowledge and provide experimental assistance. LC: conceived and designed the experiments. YaS and XS: writing—review and editing. All authors contributed to the article and approved the submitted version.

## Abbreviations

HEI-OC1: House Ear Institute-Organ of Corti 1
ARHL: age-related hearing loss
AD: Alzheimer’s disease
PD: Parkinson’s disease
ABR: Auditory-evoked brainstem response
DPOAE: Distortion-product otoacoustic emission
H&E staining: Hematoxylin and Eosin staining
SGNs: spiral ganglia neurons
SV: stria vascularis
qRT-PCR: Quantitative reverse-transcriptase polymerase chain reaction
*ANOVA*: Analysis of Variance
NF-κB: nuclear factor kappa B
PVM/Ms: perivascular macrophage-like melanocytes
BLB: blood-labyrinth barrier
EP: endcochlear potential.
